# Optimal Sensor Formation for 3D Cooperative Localization of AUVs Using Time Difference of Arrival (TDOA) Method

**DOI:** 10.3390/s18124442

**Published:** 2018-12-15

**Authors:** Xu Bo, Asghar A. Razzaqi, Xiaoyu Wang

**Affiliations:** College of Automation, Harbin Engineering University, Harbin 150001, China; xubocarter@hrbeu.edu.cn (X.B.); Wxiaoyu@hrbeu.edu.cn (X.W.)

**Keywords:** cooperative localization, autonomous underwater vehicles (AUVs), Time Difference of Arrival (TDOA), optimal formation, Fisher Information Matrix

## Abstract

The cooperative localization of submerged autonomous underwater vehicles (AUVs) using the Time Difference of Arrival (TDOA) measurements of surface AUV sensors is an effective method for many applications of AUVs. Proper positioning of the sensors to maximize the observability of the AUVs is very critical for cooperative localization. In this paper, a novel method for obtaining the optimal formation of sensor AUVs has been presented for the three-dimensional (3D) cooperative localization of targets using the TDOA technique. An evaluation function for estimating the optimal formation has been derived based on Fisher Information Matrix (FIM) theory for a single target as well as multiple-target cooperative localization systems. An iterative stepping algorithm has been followed to solve the evaluation function and obtain the optimal positions of the sensors. The algorithm ensured that the computation complexity should remain limited, even when the number of sensor AUVs is increased. Various simulation examples are then presented to calculate the optimal formation for different systems/situations. The effect of the position of the reference sensor and operating depth of the target AUVs on the optimal formation of the sensors has also been studied, and conclusions are drawn. For implementation of the proposed method for more practical scenarios, a simulation example is also presented for cases when the target’s position is only known with uncertainty.

## 1. Introduction

Autonomous underwater vehicles (AUVs) have been the focus of research in recent times due to their increasing applications in both military and civil fields such as oceanographic surveys, underwater search and rescue, underwater structure monitoring, underwater surveillance and mine counter operations, etc. Most of these applications require the localization of AUVs during the whole operation in order to ensure the accuracy of the collected data as well as the safety of the AUVs. However, compared to ground vessels, the self-localization of an underwater vessel is a much complex and challenging task due to non-availability of Global Positioning System (GPS) signals and attenuation of Radio Frequency (RF) waves in water [1]. Therefore, AUVs have to rely on an Inertial Navigation System (INS) for their underwater localization and navigation. Notwithstanding the above, even the high-accuracy inertial sensors may accumulate errors, with the passage of time causing unbounded error in the localization of the AUVs. Various techniques have been proposed and are being utilized to limit the error of INS and allow longer operations of AUVs, such as the frequent surfacing of AUVs for receiving GPS signals, bottom or water tracking using Doppler velocity log (DVL) [2,3], an acoustic baseline system using pre-deployed beacons [4,5,6], and cooperative localization. 

Cooperative localization is one of the most recent and advanced techniques being studied for the localization of AUVs. In cooperative localization, multiple AUVs operate together, out of which some of the AUVs, which are called pilot vessels or sensors, localize their position accurately by staying at the water surface and receiving a GPS feed. Meanwhile, the remaining AUVs, which are called slave vessels or targets, measure their position relative to the accurate position of the sensor AUVs and apply correction to the INS to bound its error. The key methods to estimate the target’s position using sensor AUVs’ information include the Received Signal Strength (RSS), Angle of Arrival (AoA), Range measurement and Time Difference of Arrival (TDOA). RSS and AoA may not be the most suitable choices for the localization of targets due to time-varying path loss, multiple path affect, and the bending of sound waves in water [7]. Range measurement and TDOA are more accurate and commonly used methods for the cooperative localization of AUVs. The range measurement method requires precise time clock synchronization among sensors and targets, which is a challenging task. On the other hand, the TDOA method can be implemented without clock synchronization.

**Figure 1:**
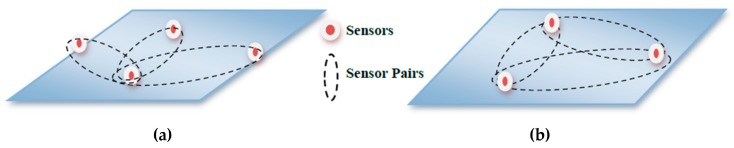
Time Difference of Arrival (TDOA) sensor pairing techniques (**a**) Centralized Sensor Pairing (CSP); (**b**) Decentralized Sensor Pairing (DSP).

The TDOA method depends on processing the time difference of the multiple signals that are generated from sensor AUVs when received at the target AUVs. Each TDOA measurement determines that the target should lie on a hyperbolic curve with a constant range difference between the two transmitting reference sensors. At least two pairs of sensors are needed to estimate the location of a target device in two dimensions (2D), and three pairs of sensors are required for its three-dimensional (3D) localization [8]. Two types of sensor pair strategies have been studied in the literature i.e., Centralized Sensor Pairing (CSP) and De-centralized Sensor Pairing (DSP). In CSP, one of the sensors is declared as a common reference, and all of the other sensors make pairs with the common reference for TDOA measurements. So, for CSP, the maximum possible number of sensor pairs are *M*−1, where *M* is the total number of sensors in the system. Meanwhile, in DSP, no sensor is declared as the common reference, and any two sensors can make a sensor pair. For DSP, the maximum possible sensor pairs is *M*(*M*−1)/2. Although CSP is a commonly used and widely studied technique due to its ease of implementation, it has an observability constraint due to power, bandwidth, and communication range limitations. Figure 1a,b explain the basic working principles of CSP and DSP, respectively.

Beside other implementation challenges, the accurate measurement of TDOA using acoustic waves is a difficult task due to high transmission loss, multipath effect, the varying speed of sound in water, and the dependence of sound propagation on bathy conditions. Although the above-mentioned factors are mostly uncontrollable, the accuracy of TDOA cooperative localization can still be optimized using a suitable estimation algorithm and by maximizing the observability of the sensor AUVs by stationing them at maximum observable locations (called the optimal formation of sensor AUVs).

While extensive research has been carried out in the field of estimation algorithm development for cooperative localization, limited attention has been paid to the problem of the optimal formation of sensor AUVs. In [9], Baccou et al. presented a method for the position estimation of an AUV by measuring its range from a fixed beacon from different locations around the beacon. AUV was navigated in a way to optimize the observability of the beacon by maximizing the Fisher Information Matrix (FIM). In [10], Xinpeng et al. proposed an evaluation function based on Cramer–Rao Lower Bound (CRLB) and FIM to estimate the optimal formation of two leaders for the localization of AUVs. It was suggested that for two leaders’ cooperative localization, the optimal formation was 90 degrees of separation between the leader and the follower AUVs. Further, by utilizing the same method, they presented the optimal formation for three leaders and one follower system in [11]. It was recommended that leaders should stay around the follower at 120 degrees in relation to each other for achieving the maximum observability. Moreover, the sensitivity of the localization performance with respect to the smaller change of distance and angle between leaders and followers was analyzed. Moreno-Salinas et al. presented a few research papers in the field of optimal formation for cooperative localization [12,13,14,15,16,17]. In [12], the problem of computing an optimal geometric configuration of surface sensors was addressed for maximizing the range-related information available for multiple targets’ localization in a three-dimensional space through using the techniques of Pareto optimization and estimation theory. In [13], the same problem was addressed, considering that the initial estimate of the target is available with uncertainty. In [14], the optimal formation of multiple leaders was studied for the cooperative localization of multiple followers with uncertainty in their position. Moreover, it was shown that the optimal formation depends on the constraints imposed on the sensor configuration, the target’s position, and the probabilistic distributions that define the prior uncertainty in each target’s position. The case of cooperative localization with bearing-only measurements was discussed in [15], and the optimal formation of leader vessels was achieved using the CRLB matrix. In [16], the optimal path for a single moving leader vessel was proposed for the localization of a stationary AUV using two different approaches; i.e., the first approach computes only the next point that the vessel should move to, while the second approach computes the complete optimal trajectory for the leader vessel. Again, in [17], the optimal formation for multiple leader vessels for multiple AUVs with uncertainty in their position was obtained using 3D range measurements only.

It can be noted that most of the research that has been conducted in the field of optimal formation for the cooperative localization of AUVs focused on the technique of relative range measurement of the targets with respect to the sensors. On the other hand, the optimal formation for cooperative localization using TDOA has not been fully explored, even though it is one of the current techniques being used for cooperative localization. It is also easy to implement, because it does not require clock synchronization between sensors and targets. In [18], the optimal sensor formation in 2D was proposed for TDOA using uncertainty minimization criteria. It was concluded with examples that for five or fewer sensors, the optimal formation is an equiangular placement of sensors around the emitter. For more than five sensors, optimal sensor placement is achieved by sensor partitions of suitable size, each with equiangular separation. Moreover, it was also concluded that the optimal formation is independent of the range of sensors from the emitter. In [19], a novel method was proposed for the optimal placement of sensors one by one in 2D using TDOA. The method focused on finding the optimal location of one sensor at a time instead of finding the optimal formation of all the sensors simultaneously. This method had the advantage of adding new sensors to the system without disturbing the already-placed sensors. A genetic algorithm was proposed for multiple objective optimal sensor placements for TDOA localization in [20]. Again, in [21], the 2D sensor placement method based on CRLB criteria was proposed for TDOA and Frequency Difference of Arrival (FDOA) localization. It was shown that the optimal configuration for this hybrid-type localization is not the simple distribution of sensors around the target in a circle; rather, the optimal configuration for this case is a combination of circular distribution with different radii. In [22], optimal sensor-target geometry was studied using CRLB and posterior error covariance for a TDOA localization problem that considered prior uncertainty in the target position. An analytical solution was found for both centralized and de-centralized TDOA sensor-pairing for both static and moving targets. Although there are some research papers available in the literature on the optimal sensor formation for TDOA-based localization in the area of sensor networks, the same problem for the cooperative localization of AUVs has rarely been studied. The cooperative localization of AUVs offers unique research challenges due to its dynamic environmental conditions. Some of the issues that need to be considered for AUVs’ localization are as follows:

1. Target AUVs are to be localized underwater in **ℜ^3^**, while the sensors of AUVs are generally placed on the surface of water in 2D. This is contrary to what was considered in most of the previous literature, where sensors and targets were considered to be placed in 2D only. In the few cases where 3D localization was considered, it was assumed that both sensors and targets can be located anywhere in 3D.
2. Another important aspect that is to be considered for underwater localization is that measurement noise is distance-dependent due to acoustic signal spreading.
3. Underwater TDOA measurements depend upon the speed of sound, which varies with the depth. Moreover, the acoustic wave does not travel in a straight path, as is the case for above water waves. However, in the literature of cooperative localization and sensor placement, the speed of sound was mostly taken as a constant, and sound wave was considered to be traveling in a straight line.

In this paper, we have analyzed the problem of finding the optimal formation of sensor AUVs for the cooperative localization of target AUVs using TDOA measurements. In contrast to previous research studies, we have considered most of the constraints that are faced in the real-world problem of the cooperative localization of AUVs. First of all, an evaluation function based on CRLB and FIM has been constructed, keeping in view these constraints. Then, the evaluation function has been used to find the optimal formation of the sensor AUVs using a recursive optimization strategy. Simulation examples for different scenarios have also been presented to demonstrate the efficacy of the proposed method for obtaining optimal sensor formation.

The remaining paper is organized as follows. Problem formulation is affirmed in the next section. The proposed evaluation function based on FIM and CRLB is derived in Section 3. The optimal solution of the evaluation function based on recursive strategy has been explained in Section 4. In Section 5, various simulation examples are presented for different scenarios, and the paper is concluded in Section 6.

## 2. Problem Formulation

Consider a target AUV operating underwater at known depth $Z_{}$. In order to localize the target AUV using the TDOA measurement technique, N sensor AUVs are deployed on the surface. 

Let $t_{ij}=t_{i}-t_{j}$ be the measured time difference of arrival between the *i*th and *j*th sensors. The measured TDOA vector is given by:

$$\tilde{t}=\left[\begin{array}{c} t_{ij}\\ \vdots \\ \vdots \end{array}\right]$$

where {ij}∈Γ and Γ depend upon the type of sensor pairing used for TDOA. The actual range-difference vector is given by:

$$d=\left[\begin{array}{c} d_{ij}\\ \vdots \\ \vdots \end{array}\right]$$

where $d_{ij}=d_{i}-d_{j}$ is the range difference between the *i*th and *j*th sensors and is given by:

$$d_{ij}=||p_{i}-q||-||p_{j}-q||$$

where $p_{i}$ and q are the positions of the *i*th sensor and the target, respectively, and are given by:

$$p_{i}={\left[x_{i},\;y_{i}\right]}^{T}\;\mathrm{and}\;q={\left[x,\;y\;\right]}^{T}\;\mathrm{for}\;2D$$

$$p_{i}={\left[x_{i},\;y_{i},\;z_{i}\right]}^{T}\;\mathrm{and}\;q={\left[x,\;y,\;z\right]}^{T}\;\mathrm{for}\;3D$$

Keeping in view the characteristics of acoustic signal propagation in water, we assume that the measured time differences will be corrupted by measurement noise, which is dependent on how far the two sensors are located from the target. The noise signal may be defined as [17]:

(1) $$w_{ij}=\sigma \left(1+\eta d_{ij}{}^{\gamma }\right)$$

where σ is measurement noise constant and η and γ are the constant modelling parameters related to distance. Let measurement noise vector is given as:

(2) $$w=\left[\begin{array}{c} w_{ij}\\ \vdots \\ \vdots \end{array}\right]$$

where, {ij}∈Γ. Then, covariance matrix of measurement noise can be obtained as follows:

(3) $$E\left(w\cdot w^{T}\right)=\Sigma =\sigma^{2}{\left(1+\eta d_{ij}{}^{\gamma }\right)}^{2}\cdot I$$

where I is an *n*-by-*n* identity matrix.

In most of the previous work on optimal formation, the underwater sound speed was taken as a constant, although it varies with temperature, pressure, and salinity. Moreover, it was also assumed that the sound wave propagates in a straight line, and hence the time of flight was taken as:

$$t=\frac{d}{C}$$

**Figure 2:**
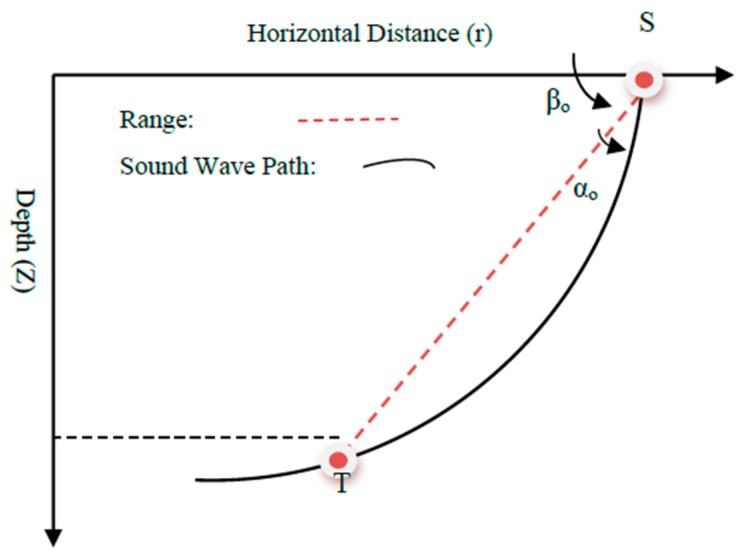
Curved path followed by the sound wave from the sensor to the target autonomous underwater vehicle (AUV).

Both of the assumptions are clearly far from practical scenarios, and may induce error in the final results. In order to consider these important aspects, we have assumed that the underwater Sound Speed Profile (SSP) is a function of the depth, and the sound wave travels along a curved path, as shown in Figure 2 [23]. For our model of underwater sound propagation, we assume the following:

- A direct propagation path exists between the sensors and the target in the presence of a typical underwater multipath environment.
- It is possible to pick up and identify the first received wave to measure the time of arrival.
- The SSP is known, and the sound speed is only depth-dependent (isogradient) i.e., the field of interest is vertically stratified.

With the above assumptions, the speed of sound is given as [23,24]:

(4) v(z)=az+b

where z indicates the depth of the target AUV, a is the steepness of the SSP, and b represents the sound speed at the water surface. 

The time of flight of the sound wave from the sensor to the target along the curved path is calculated as follows [23,25]:

(5) $$t_{i}=-\frac{1}{a}\left(\ln\frac{1+\sin\theta_{i}}{\cos\theta_{i}}-\ln\frac{1+\sin\theta_{t}}{\cos\theta_{t}}\right)$$

where $\theta_{i}$ and $\theta_{t}$ are the sound wave angles at the *i*th sensor and the target, respectively, and are given as:

$$\theta_{i}=\beta_{o}+\alpha_{o}$$

$$\theta_{t}=\beta_{o}-\alpha_{o}$$

where $\alpha_{o}$ and $\beta_{o}$ are defined in Figure 2 and are calculated as:

$$\tan\beta_{o}=\frac{z-z_{i}}{r-r_{i}}$$

$$\frac{1-\tan\beta_{o}\tan\alpha_{o}}{1+\tan\beta_{o}\tan\alpha_{o}}=\frac{b+az}{b+az_{i}}$$

where z and $z_{i}$ are the operating depths of the target and the *i*th sensor, respectively. Moreover, $r-r_{i}$ indicates the horizontal distance between the target and the *i*th sensor, and is defined as:

(6) $$r-r_{i}=\sqrt{{\left(x-x_{i}\right)}^{2}+{\left(y-y_{i}\right)}^{2}}$$

## 3. Derivation of Evaluation Function

In this section, we have used the theory of CRLB and FIM to derive the proposed evaluation function for estimating the optimal formation. In first step, we will derive the evaluation function for a single-target cooperative localization system. Then, based on its outcome, we will derive the evaluation function for a multiple-target system.

### 3.1. Single-Target Cooperative Localization

The maximum likelihood function for $\tilde{t}$, given the target position q and noise covariance matrix Σ, is defined as [26]:

(7) $$P\left(\tilde{t}\right)=\frac{1}{{\left(2\pi \right)}^{\frac{n}{2}}{\left|\Sigma_{j}\right|}^{\frac{1}{2}}}\exp\left\{-\frac{1}{2}{\left(\tilde{t}-t\right)}^{T}\Sigma^{-1}\left(\tilde{t}-t\right)\right\}$$

By definition of FIM [27]:

(8) $$FIM=E\left\{\left(\frac{\partial }{\partial q}\ln\left(P\left(\tilde{t}\right)\right)\right)\cdot {\left(\frac{\partial }{\partial q}\ln\left(P\left(\tilde{t}\right)\right)\right)}^{T}\right\}$$

where E{.} denotes the expected value. Taking the natural logarithm of Equation (7), we get:

(9) $$\ln\left(P\left(\tilde{t}\right)\right)=Const-\frac{1}{2}{\left(\tilde{t}-t\right)}^{T}\Sigma^{-1}\left(\tilde{t}-t\right)$$

where $Const=-\frac{1}{2}\left(n\cdot \ln(2\pi )+\ln\left(\left|\Sigma_{j}\right|\right)\right)$.

After taking the partial derivative of Equation (9) with respect to (w.r.t.) q, we will get:

(10) $$\frac{\partial }{\partial q}\ln\left(P\left(\tilde{t}\right)\right)=\frac{\partial }{\partial q}t^{T}\Sigma^{-1}\left(\tilde{t}-t\right)$$

Now, we will calculate the derivative $\frac{\partial }{\partial q}t^{T}$ for Equation (10). Taking the derivative of Equation (5) w.r.t r and z, we get [23]:

(11) $$\begin{array}{l} \frac{\partial t_{i}}{\partial r}=-\frac{1}{a}\left(\frac{1}{\cos\theta_{t}{}^{}}\frac{\partial \theta_{t}}{\partial r}-\frac{1}{\cos\theta_{i}{}^{}}\frac{\partial \theta_{i}}{\partial r}\right)\\ \frac{\partial t_{i}}{\partial z}=-\frac{1}{a}\left(\frac{1}{\cos\theta_{t}{}^{}}\frac{\partial \theta_{t}}{\partial z}-\frac{1}{\cos\theta_{i}{}^{}}\frac{\partial \theta_{i}}{\partial z}\right) \end{array}$$

$$\frac{\partial }{\partial q}t_{i}={\left[\begin{array}{c} \frac{\partial t_{i}}{\partial r}\\ \frac{\partial t_{i}}{\partial z} \end{array}\right]}^{T}$$

(12) $$\frac{\partial }{\partial q}t={{\left[\begin{array}{c} \frac{\partial t_{i}}{\partial r}-\frac{\partial t_{j}}{\partial r}\\ \frac{\partial t_{i}}{\partial z}-\frac{\partial t_{j}}{\partial z} \end{array}\right]}^{T}}_{\{i,j\}\in \Gamma }=D$$

Similarly:

(13) $$\frac{\partial }{\partial q}t^{T}=D^{T}$$

Putting the values from Equations (12) and (13) to Equation (10), we get:

(14) $$\frac{\partial }{\partial q}\ln\left(P\left(\tilde{t}\right)\right)=D^{T}\Sigma^{-1}\left(\tilde{t}-t\right)$$

Combining Equation (14) and Equation (8):

$$FIM=E\left[D^{T}\Sigma^{-1}\left(\tilde{t}-t\right){\left(\tilde{t}-t\right)}^{T}\Sigma^{-1}D\right]$$

(15) $$FIM=D^{T}\Sigma^{-1}D$$

Equation (15) gives the general form of FIM for TDOA cooperative localization. In the next step, we will compute the matrix D for both types of sensor pairing, i.e., CSP and DSP.

#### 3.1.1. Centralized Sensor Pairing (CSP)

CSP is the most commonly used method of sensor pairing for TDOA measurements. Let’s assume that there are n sensors, and the sensor-1 is taken as the reference sensor; then, {i,j} for this case will become {(2,1), (3,1), …, (n,1)}. The matrix D as given in Equation (12) is now defined as:

$$D=\left[\begin{array}{cc} \frac{\partial t_{2}}{\partial r}-\frac{\partial t_{1}}{\partial r} & \frac{\partial t_{2}}{\partial z}-\frac{\partial t_{1}}{\partial z}\\ \frac{\partial t_{3}}{\partial r}-\frac{\partial t_{1}}{\partial r} & \frac{\partial t_{3}}{\partial z}-\frac{\partial t_{1}}{\partial z}\\ . & .\\ . & .\\ . & .\\ \frac{\partial t_{n}}{\partial r}-\frac{\partial t_{1}}{\partial r} & \frac{\partial t_{n}}{\partial z}-\frac{\partial t_{1}}{\partial z} \end{array}]=[\begin{array}{ccc} \frac{\partial t_{2}}{\partial x}-\frac{\partial t_{1}}{\partial x} & \frac{\partial t_{2}}{\partial y}-\frac{\partial t_{1}}{\partial y} & \frac{\partial t_{2}}{\partial z}-\frac{\partial t_{1}}{\partial z}\\ \frac{\partial t_{3}}{\partial x}-\frac{\partial t_{1}}{\partial x} & \frac{\partial t_{3}}{\partial y}-\frac{\partial t_{1}}{\partial y} & \frac{\partial t_{3}}{\partial z}-\frac{\partial t_{1}}{\partial z}\\ . & . & .\\ . & . & .\\ . & . & .\\ \frac{\partial t_{n}}{\partial x}-\frac{\partial t_{1}}{\partial x} & \frac{\partial t_{n}}{\partial y}-\frac{\partial t_{1}}{\partial y} & \frac{\partial t_{n}}{\partial z}-\frac{\partial t_{1}}{\partial z} \end{array}\right]$$

As all of the sensors are supposed to be placed at surface (depth = 0), the derivatives with respect to the z-coordinates of the target position are not required, and may be dropped. So:

$$D=\left[\begin{array}{cc} \frac{\partial t_{2}}{\partial x}-\frac{\partial t_{1}}{\partial x} & \frac{\partial t_{2}}{\partial y}-\frac{\partial t_{1}}{\partial y}\\ \frac{\partial t_{3}}{\partial x}-\frac{\partial t_{1}}{\partial x} & \frac{\partial t_{3}}{\partial y}-\frac{\partial t_{1}}{\partial y}\\ . & .\\ . & .\\ . & .\\ \frac{\partial t_{n}}{\partial x}-\frac{\partial t_{1}}{\partial x} & \frac{\partial t_{n}}{\partial y}-\frac{\partial t_{1}}{\partial y} \end{array}\right]$$

So, FIM for CSP is now defined as:

(16) $$FIM=D^{T}\Sigma^{-1}D=\left[\begin{array}{cc} \sum_{i=2}^{n}\frac{a_{ix}^{2}}{\Sigma_{i}} & \sum_{i=2}^{n}\frac{a_{ix}^{}.a_{iy}^{}}{\Sigma_{i}}\\ \sum_{i=2}^{n}\frac{a_{iy}^{}.a_{ix}^{}}{\Sigma_{i}} & \sum_{i=2}^{n}\frac{a_{iy}^{2}}{\Sigma_{i}} \end{array}\right]$$

where:

$$a_{ix}=\frac{\partial t_{i}}{\partial x}-\frac{\partial t_{1}}{\partial x},a_{iy}=\frac{\partial t_{i}}{\partial y}-\frac{\partial t_{1}}{\partial y}\;\mathrm{and}\;\Sigma_{i}=\sigma^{2}{\left(1+\eta d_{i1}{}^{\gamma }\right)}^{2}$$

The determinant of the *FIM* can be found as follows:

(17) $$\left|FIM\right|=\left[\sum_{i=2}^{n}\frac{a_{ix}^{2}}{\Sigma_{i}}.\sum_{i=2}^{n}\frac{a_{iy}^{2}}{\Sigma_{i}}-{\left(\sum_{i=2}^{n}\frac{a_{ix}^{}.a_{iy}^{}}{\Sigma_{i}}\right)}^{2}\right]$$

The determinant of the *FIM*, as defined by Equation (17), represents the observability of the target AUV. When a sensor AUV changes its position, the determinant of the *FIM* will be changed, and hence, the observability will also be changed. When the determinant will reach its maximum, the corresponding formation will be the optimal formation of the sensor AUVs. So, for the single target cooperative localization system, Equation (17) will be used as the evaluation function for estimating the optimal positions of sensor AUVs.

#### 3.1.2. De-Centralized Sensor Pairing

Due to power, bandwidth, and observability constraints, sometimes, centralized sensor pairing may not be realistic. Hence, de-centralized sensor pairing is also considered, which can handle the above-mentioned issues. DSP also reduces the minimum number of sensors that is required for cooperative localization. For *n* sensors, a maximum of *n*(*n*−1)/2 sensor pairs are possible in DSP. So, in the case of DSP, only three sensors are required instead of four sensors for obtaining three TDOA measurements.

Here, we consider an example of three sensor AUVs, i.e., n=3. Then, {i,j} will be {(1,2), (1,3), (2,3)}. The matrix D as given Equation (12) is now defined as:

$$D=\left[\begin{array}{cc} \frac{\partial t_{1}}{\partial x}-{\frac{\partial t_{2}}{\partial x}}_{} & \frac{\partial t_{1}}{\partial y}-{\frac{\partial t_{2}}{\partial y}}_{}\\ \begin{array}{l} \frac{\partial t_{1}}{\partial x}-\frac{\partial t_{3}}{\partial x}\\ \frac{\partial t_{2}}{\partial x}-\frac{\partial t_{3}}{\partial x} \end{array} & \begin{array}{l} \frac{\partial t_{1}}{\partial y}-\frac{\partial t_{3}}{\partial y}\\ \frac{\partial t_{2}}{\partial y}-\frac{\partial t_{3}}{\partial y} \end{array} \end{array}\right]$$

So, the FIM for DSP with three sensor AUVs is now defined as:

(18) $$FIM=[\begin{array}{cc} \frac{a_{12x}^{2}}{\Sigma_{12}}+\frac{a_{13x}^{2}}{\Sigma_{13}} & \frac{a_{12x}^{}\cdot a_{12y}^{}}{\Sigma_{12}}+\frac{a_{13x}^{}\cdot a_{13y}^{}}{\Sigma_{13}}\\ +\frac{a_{23x}^{2}}{\Sigma_{23}} & +\frac{a_{23x}^{}\cdot a_{23y}^{}}{\Sigma_{23}}\\ \frac{a_{12y}^{}\cdot a_{12x}^{}}{\Sigma_{12}}+\frac{a_{13y}^{}\cdot a_{13x}^{}}{\Sigma_{13}} & \frac{a_{12y}^{2}}{\Sigma_{12}}+\frac{a_{13y}^{2}}{\Sigma_{13}}\\ +\frac{a_{23y}^{}\cdot a_{23x}^{}}{\Sigma_{23}} & +\frac{a_{23y}^{2}}{\Sigma_{23}} \end{array}]$$

where:

$$a_{ijx}=\frac{\partial t_{i}}{\partial x}-\frac{\partial t_{j}}{\partial x},a_{ijy}=\frac{\partial t_{i}}{\partial y}-\frac{\partial t_{j}}{\partial y}\;\mathrm{and}:\;\Sigma_{ij}=\sigma^{2}{\left(1+\eta d_{ij}{}^{\gamma }\right)}^{2}$$

After calculating the FIM using Equation (18), we will calculate its determinant and then solve it for the maximum value, in order to obtain the optimal sensor positions, which is similar to what we have done in the CSP case.

### 3.2. Multiple Targets Cooperative Localization

In order to derive the evaluation function for a multiple-targets cooperative localization system, we will first calculate Equation (17) separately for each target as follows:

(19) $$FIM(j)=D_{j}{}^{T}\Sigma^{-1}D_{j}$$

$$D_{j}=\left[\begin{array}{cc} \frac{\partial t_{2}}{\partial x_{Tj}}-\frac{\partial t_{1}}{\partial x_{Tj}} & \frac{\partial t_{2}}{\partial y_{Tj}}-\frac{\partial t_{1}}{\partial y_{Tj}}\\ \frac{\partial t_{3}}{\partial x_{Tj}}-\frac{\partial t_{1}}{\partial x_{Tj}} & \frac{\partial t_{3}}{\partial y_{Tj}}-\frac{\partial t_{1}}{\partial y_{Tj}}\\ . & .\\ . & .\\ . & .\\ \frac{\partial t_{n}}{\partial x_{Tj}}-\frac{\partial t_{1}}{\partial x_{Tj}} & \frac{\partial t_{n}}{\partial y_{Tj}}-\frac{\partial t_{1}}{\partial y_{Tj}} \end{array}\right]$$

where j=1,2,…m, and m is the total number of target AUVs. Moreover, subscript “*Tj*” indicates the coordinates of the *j*th target AUV. The corresponding *FIM* for each target will now become:

(20) $$FIM(j)=\left[\begin{array}{cc} \sum_{i=2}^{n}\frac{a_{ijx}^{2}}{\Sigma_{i}} & \sum_{i=2}^{n}\frac{a_{ijx}^{}\cdot a_{ijy}^{}}{\Sigma_{i}}\\ \sum_{i=2}^{n}\frac{a_{ijy}^{}\cdot a_{ijx}^{}}{\Sigma_{i}} & \sum_{i=2}^{n}\frac{a_{ijy}^{2}}{\Sigma_{i}} \end{array}\right]$$

where:

$$a_{ijx}=\frac{\partial t_{i}}{\partial x_{Tj}}-\frac{\partial t_{1}}{\partial x_{Tj}},a_{ijy}=\frac{\partial t_{i}}{\partial y_{Tj}}-\frac{\partial t_{1}}{\partial y_{Tj}}\;\mathrm{and}\;\Sigma_{i}=\sigma^{2}{\left(1+\eta d_{i1}{}^{\gamma }\right)}^{2}$$

After calculating the *FIM(j)* for each target, its determinant will be calculated as |FIM(j)|. The determinant of the *FIM* for each target AUV, (|FIM(j)|), represents its observability. When a sensor AUV changes its position, the determinant of a given target AUV will be changed, and hence, its observability will also be changed. When the determinant reaches its maximum, the corresponding position will be the optimal position of that sensor with respect to the given target AUV. However, this position may not be the optimal for another target AUV. So, in order to derive the comprehensive optimal formation for all the targets simultaneously, we select the sum of the logarithm of the FIM determinant as the evaluation function *F.* The function is given by:

(21) $$F=\sum_{j=1}^{m}\ln\left(\left|FIM\left(q_{j}\right)\right|\right)$$

When the evaluation function, which is given by Equation (21), reaches its maximum, the corresponding formation of the sensor AUVs will be in an optimal formation.

### 3.3. Uncertainty in the Targets’ Location

So far, we have considered that the target position is precisely known even before the placement of the sensors to their optimal position. It clearly defeats the purpose of formulating a method for estimating the target’s position, as it is already known in advance. In practical scenarios, the target’s position is only known with uncertainty in the xy plane, while its depth is precisely known using a depth sensor. In order to solve such a problem, we consider that the target is known to lie in a well-defined region of uncertainty $D_{j}$, which is described in terms of the probability density function $f\left(q_{j}\right)$. It is to be noted that $f\left(q_{j}\right)$ will depend upon the type of operation that is being conducted by the target AUVs. For example, if the target is mostly operating in the center of a given region, $f\left(q_{j}\right)$ will be Gaussian distribution centred on a defined point. On the other hand, if only the operation area of the target is known, $f\left(q_{j}\right)$ will be uniformly distributed in that area. 

Let $D_{j}$ be the possible distribution area of the *j*th target and $f\left(q_{j}\right)$ be its PDF; then, we can get the required evaluation function as follows:

(22) $$F=\sum_{j=1}^{m}\ln\left(\int_{D_{j}}\left|FIM\left(q_{j}\right)\right|\cdot f\left(q_{j}\right)dq_{j}\right)$$

**Figure 3:**
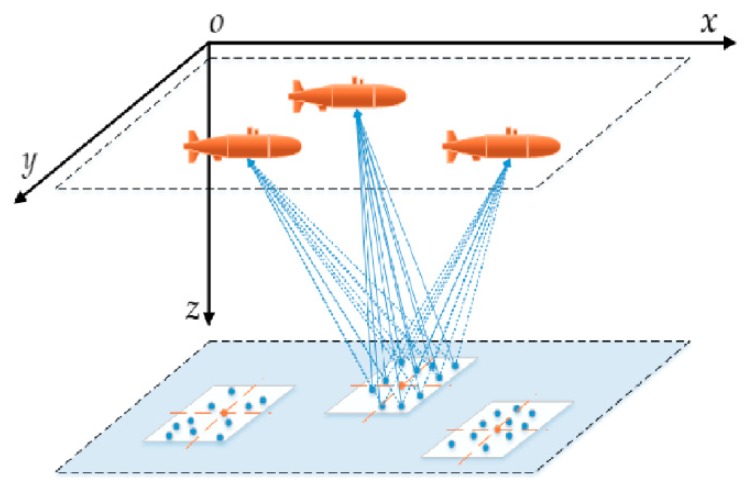
Description of the targets with uncertainty in position.

Considering the complexity of the calculation of Equation (22), we use the following methodology to solve the evaluation function:

- We assumed that the Probability Density Function (PDF) of the *j*th target is a uniform distribution within a 10×10 square area centered on $\left(x_{Tj},y_{Tj}\right)$, and the PDF is defined as follows:
  (23) $$\begin{array}{l} f\left(x_{Tj}\right)=\frac{1}{10}\;(x_{Tj}-5<x<x_{Tj}+5)\\ f\left(y_{Tj}\right)=\frac{1}{10}\;(y_{Tj}-5<y<y_{Tj}+5) \end{array}$$
- Based on the Monte-Carlo method, we built a sampling function with uniformly distributed input. By randomly selecting number r from R(0,1) and putting it in ξ=f(r), we obtain 100 possible values in the range of (a,b) as follows:
  ξ=a+(b−a)r
- As shown in Figure 3, two sets of 10 random numbers, satisfying the uniform distribution within (a,b), are sequentially generated to constitute the position of the targets. The FIM of each target relative to each sensor is calculated, and the determinant of the matrix is summed and averaged to obtain $\left|FIM\left(q_{j}\right)\right|$ for Equation (21).

A simulation example of 3 + 1 sensors and one target with position uncertainty is illustrated in Section 5.3.

## 4. Iterative Stepping Algorithm

For a multiple sensor–multiple target cooperative localization system, when the number of sensors increases, the corresponding unknown quantity in Equation (21) also increases. As a result, it drastically increases the computation complexity to solve the evaluation function. In order to address this problem, an iterative stepping algorithm has been used to ensure that the calculation is achievable. The iterative stepping algorithm is implemented as follows:

- For each target AUV, calculate *FIM(j)* based on all of the sensor AUVs’ present positions ($\left(x_{i}[t],y_{i}[t]\right)$. Calculate the determinant of the FIM, |FIM(j)|, in order to get the evaluation in current step as $E_{f}[t]$.
- Calculate the derivative of the current evaluation function $E_{f}[t]$ with respect to the “x” and “y” coordinates of each sensor AUV. Denote the derivatives as $\partial E_{f}[t]/\partial x_{i}$ and $\partial E_{f}[t]/\partial y_{i}$.
- Update the position of each sensor AUV as follows:
  (24) $$x_{i}[t+1]=x_{i}[t]+S.\frac{\partial E_{f}[t]}{\partial x_{i}[t]}$$
  (25) $$y_{i}[t+1]=y_{i}[t]+S.\frac{\partial E_{f}[t]}{\partial y_{i}[t]}$$
  where *S* is the step size for each calculation step, and is taken as 100.
- Calculate *FIM(j)* based on the updated sensor positions $\left(x_{i}[t+1],y_{i}[t+1]\right)$, and subsequently calculate the evaluation function as $E_{f}[t+1]$.
- Compare the present step evaluation function to the previous step evaluation. If $E_{f}[t+1]>E_{f}[t]$, it means that the updated sensors’ positions can improve the observability of the system. So, go back to step (1) to start the next calculation based on the updated sensors’ positions. If $E_{f}[t+1]<E_{f}[t]$, it means that the current sensors’ positions are optimal for cooperation localization, as the system observability is at its maximum. The algorithm is to be terminated.

## 5. Simulation Examples

In this section, a few simulation examples of estimating the optimal formation for different scenarios are presented using the proposed method. Simulation examples for single-target and multiple-targets cooperative localization systems are presented in the ensuing paragraphs:

### 5.1. Single-Target Cooperative Localization System

In this section, a simple example of one target AUV is simulated for obtaining the optimal formation of the sensors. All of the sensors are supposed to be operating on a surface at zero depths, while the target AUV is assumed at depths of 50 m, 75 m, and 100 m for different examples. The sound speed at surface (b) and the steepness of SSP (a) in Equation (4) are taken as 1500 and 0.1, respectively. Moreover, the values of parameters σ, η, and γ in Equation (3) are taken as 0.1, 0.05, and 1, respectively.

#### 5.1.1. Centralized Sensor Pairing

For single-target localization, four sensors are considered in this example with centralized sensor pairing. The reference sensor (sensor 1) is assumed to be placed along the x-axis at coordinates [25, 0, 0], and the remaining three sensors (sensors 2–4) are initially placed at random positions of [0, 40, 0], [−20, 20, 0], and [−10, −35, 0], respectively. The target AUV is assumed to be placed on the z-axis at [0, 0, −50]. The obtained location coordinates of the sensor AUVs for optimal formation are summarized in Table 1.

**Figure 4:**
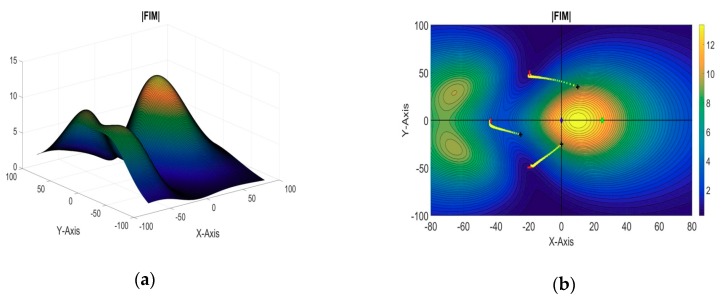
(**a**) Three-dimensional (3D) plot of the evaluation function (single target: CSP); (**b**) Two-dimensional (2D) contour plot of the evaluation function (single target: CSP).

Figure 4a shows the evaluation function plotted in a 3D map against the xy coordinates. While in Figure 4b, the evaluation function has been shown on a contour plot against the xy coordinates. The blue dot represents the two-dimensional projection position of the target AUV; the green dot represents the position of the reference sensor in the xy plane; the black squares represent the two-dimensional projection of the starting positions of the sensors, and the red crosses represent their final optimal positions. The yellow crosses show the path for the optimal sensor positions from their initial positions. We can see that the search step is coarse in the beginning of the search. However, as the search progresses, the search step decreases, and becomes very fine. This will ensure that the accuracy of the estimated positions is very high.

We have also studied the effect of the position of the reference sensor on the optimal positions of the sensor AUVs. For this purpose, we have placed the reference sensor at 25 m, 50 m, and 75 m along the x-axis, and obtained the optimal formations for each case. The optimal formations against the different positions of the reference sensor are summarized in Table 2. We can see from the table that as the reference sensor is moved closer to the target AUV, the optimal positions of the sensors becomes farther, and vice versa.

#### 5.1.2. De-Centralized Sensor Pairing

Three sensor AUVs are assumed in this example, and the DSP pairing of the sensors is used for the TDOA calculation. So, the three sensor pairs are [(1,2), (1,3), (2,3)]. At the start of the algorithm, sensors are placed symmetrically around the target at [0, 60, 0], [−25, −20, 0], and [50, −40, 0]. The target AUV is assumed to be placed along the z-axis at various depths. In order to study the effect of target depth on the optimal formation of sensors, we have used target depths as 25 m, 50 m, and 75 m, while keeping other parameters constant. The obtained optimal formations for the DSP example are summarized in Table 3. It can be seen from the table that as the target depth is reduced, the optimal positions of the sensors become closer to the target, and vice versa. The same effect is also observed for the CSP example.

**Figure 5:**
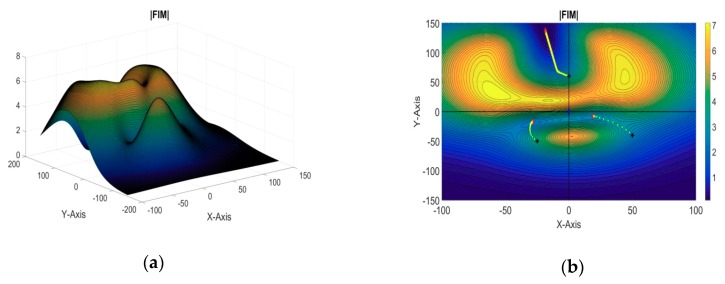
(**a**) 3D plot of the evaluation function (single target: DSP); (**b**) 2D contour plot of the evaluation function (single target: DSP).

A 3D plot of the evaluation function and its 2D contour plot against the xy plane are shown in Figure 5a,b, respectively. The blue dot represents the two-dimensional projection of the target position; the black squares represent the initial positions of the sensors, and the red crosses represent their optimal positions obtained from the algorithm.

### 5.2. Multiple-Targets Cooperative Localization System

In order to validate the efficacy of the proposed evaluation function, *F*, for the multiple-targets cooperative localization system, several simulation examples are presented in this section. All of the target AUVs in these examples are assumed to be operating at a fixed depth of 50 m.

#### 5.2.1. 3 + 1 Sensors—2 Targets

**Figure 6:**
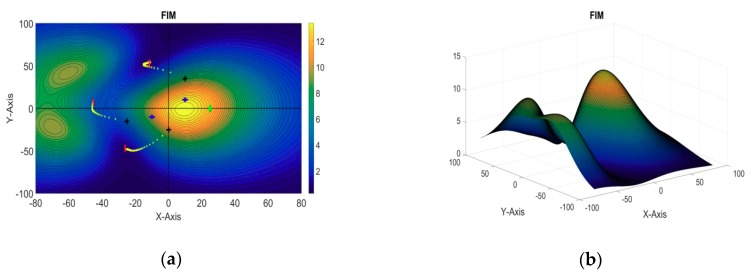
(**a**) 2D contour plot of the evaluation function (3 + 1 sensors, two targets); (**b**) 3D plot of the evaluation function (3 + 1 sensors, two targets).

In this simulation example, there are a total four sensors, out of which sensor-1 was taken as the reference sensor and the optimal formation of the remaining three sensors is estimated. The target AUVs are assumed to be operating at [−10, −10, −50] and [10, 10, −50], while the reference sensor AUV is assumed to be placed along the x-axis at [25, 0, 0]. The remaining three sensors are initially located at [10, 35, 0], [−25, −15, 0], and [0, −25, 0], respectively. 2D and 3D plots of the evaluation function against the xy plane are shown in Figure 6a,b respectively. The obtained optimal positions of the sensor AUVs are summarized in Table 4.

#### 5.2.2. 4 + 1 Sensors: Three Targets

**Figure 7:**
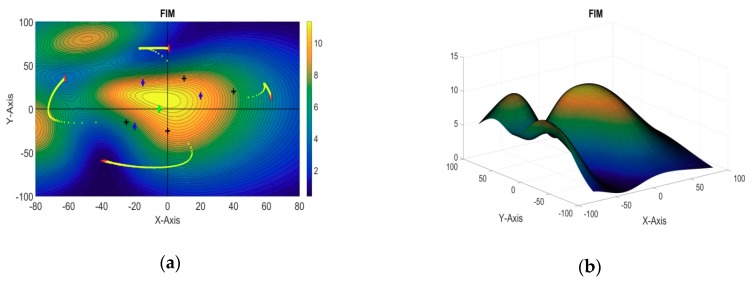
(**a**) 2D contour plot of the evaluation function (4 + 1 sensors, three targets); (**b**) 3D plot of the evaluation function (4 + 1 sensors, three targets).

In this example, the optimal formation of four sensors is estimated for a three-target cooperative localization system, while the remaining fifth sensor is taken as the reference sensor. The target AUVs are assumed to be operating at [−20, −20, −50], [−15, 30, −50], and [20, 15, −50], while the reference sensor AUV is assumed to be placed along the x-axis at [−5, 0, 0]. The initial positions of the sensor AUVs for the stepping algorithm are taken as [10, 35, 0], [−25, −15, 0], [40, 20, 0], and [0, −25, 0], respectively. The optimal positions of the sensors are summarized in Table 4. A 3D plot of the evaluation function and its 2D contour plot against the xy plane are shown in Figure 7a,b, respectively.

#### 5.2.3. 5 + 1 Sensors: Three Targets

**Figure 8:**
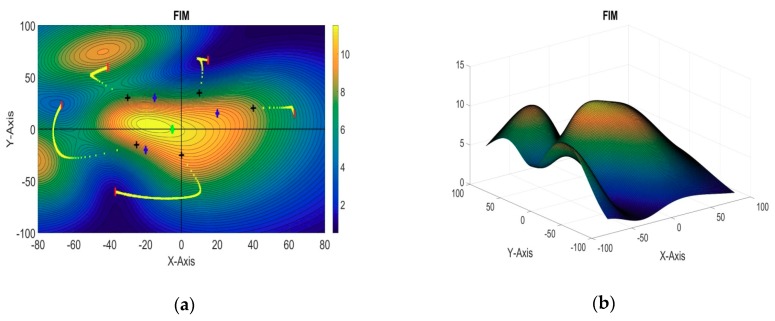
(**a**) 2D contour plot of the evaluation function (5 + 1 sensors, three targets); (**b**) 3D plot of the evaluation function (5 + 1 sensors, three targets).

In this section, the last simulation example is presented for six sensors and three targets in a cooperative localization system. The positions of the target AUVs, reference sensor, and initial positions of the first four sensors are taken to be the same as those in the previous example, while the fifth sensor is initially placed at [−30, 30, 0]. The optimal positions of the sensors obtained from the iterative algorithm are summarized in Table 4. A 3D plot of the evaluation function and its 2D contour plot against the xy plane are shown in Figure 8a,b, respectively.

### 5.3. Cooperative Localization Example with Uncertainty in Target Locaion

**Figure 9:**
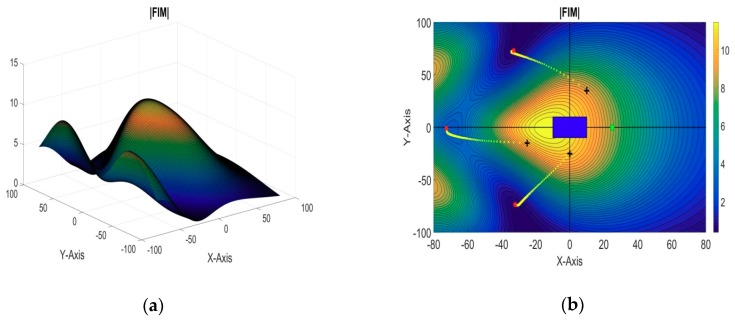
(**a**) 3D plot of the evaluation function (with uncertain target position); (**b**) 2D contour plot of the evaluation function (with uncertain target position).

In order to validate the efficacy of the proposed method in Section 3.3 for optimal sensor placement when the target position is known with uncertainty, a simulation example of a single target and 3 + 1 sensors is presented. The target is known to be located in a square of 10 m × 10 m centred on the z-axis at a 50-m depth. All of the remaining parameters are assumed to be the same as were taken in first example in Section 5.1.1. The reference sensor (sensor 1) is assumed to be placed along the x-axis at coordinates [25, 0, 0], and the remaining three sensors (sensors 2–4) are initially placed at random positions of [0, 40, 0], [−20, 20, 0], and [−10, −35, 0], respectively. The obtained location coordinates of the sensor AUVs for optimal formation are summarized in Table 5. The evaluation function plotted in a 3D map and in a 2D contour plot against the xy coordinates are shown in Figure 9a,b respectively. The blue square represents the two-dimensional projection of the target’s position uncertainty area. We can see from Figure 9b and Table 5 that the sensor formation is now spread out as compared to the similar example in Section 5.1.1 when the target’s position was precisely known.

## 6. Conclusions

A novel method for obtaining the optimal formation of sensor AUVs for the cooperative localization of submerged AUVs using TDOA measurements is presented in this paper. The effect of distance-dependent measurement noise, variable speed of sound in water, and travel of a sound wave along a curved path instead of a straight line have been considered while deriving the evaluation function. Simulation examples for obtaining the optimal formation have been presented for single-target and multiple-targets cooperative localization systems. Moreover, different types of sensor pairing in the TDOA method have also been discussed with simulation examples. A more practical scenario, when the target position is only known with uncertainty, has also been discussed along with a simulation example. Examples demonstrated applications of the methodology in a number of scenarios other than cooperative localization as well; these included cooperative sensor motion control, target tracking, adaptive control, etc. The effect of target AUVs’ operating depth and the position of the reference sensor have also been studied to illustrate how optimal formation is affected by the positions of targets as well as the reference sensor. The proposed method, when utilized for the cooperative localization of AUVs, will minimize the positioning error by maximizing the TDOA-related information. Future work will be focused on studying the performance of the proposed method when used with selected algorithms for various applications.

## Figures and Tables

**Table 1:** Optimal Positions of Sensor AUVs (Single Target: CSP).

Sensor AUVs	Optimal Positions	
	X-Coordinate	Y-Coordinate
Sensor 2	−20.02	49.73
Sensor 3	−43.55	0
Sensor 4	−20.02	−49.73

**Table 2:** Optimal Positions of Sensor AUVs (Single Target: CSP).

Reference Sensor Position	Optimal Positions		
	Sensor 1	Sensor 2	Sensor 3
[25, 0]	[−20.02, 49.73]	[−43.55, 0]	[−20.02, −49.73]
[50, 0]	[−15.16, 45.35]	[−39.90, 0]	[−15.16, −45.35]
[75, 0]	[−12.88, 42.06]	[−34.49, 0]	[−12.88, −42.06]

**Table 3:** Optimal Positions of Sensor AUVs (Single Target: DSP) and Effect of Target Depth on Optimal Formation.

Target Depth	Optimal Positions		
	Sensor 1	Sensor 2	Sensor 3
25 m	[−18.61, 137.69]	[−27.97, −16.20]	[18.82, −7.91]
50 m	[−29.98, 170.14]	[−54.10, −31.23]	[34.75, −11.22]
75 m	[−37.78, 201.37]	[−76.6, −48.93]	[47.67, −21.95]

**Table 4:** Optimal Positions of Sensor AUVs (Various Sensor–Target Configurations).

Sensor–Target Configuration	Estimated Optimal Positions				
	Sensor 1	Sensor 2	Sensor 3	Sensor 4	Sensor 5
3 + 1 sensors; 2 targets	−11.74, 53.10	−44.35, 7.98	−26.71, −46.17	x	x
4 + 1 sensors; 3 targets	−0.58, 69.24	−62.19, 35.40	−38.60, −59.42	62.99, 12.86	x
5 + 1 sensors; 3 targets	14.90, 66.32	−66.86, 23.32	−36.91, −60.39	62.96, 14.50	−41.02, 59.55

**Table 5:** Optimal Positions of Sensor AUVs (with Uncertain Target Position).

Sensor AUVs	Optimal Positions	
	X-Coordinate	Y-Coordinate
Sensor 2	−20.02	49.73
Sensor 3	−43.55	0
Sensor 4	−20.02	−9.73

## References

[1] Burdic W.S. (2002). Underwater Acoustic System Analysis.

[2] Shi W.T. (2010). DSP-Based Embedded Control System Design of Underwater Vehicle. Master’s Thesis.

[3] Yu C. (2010). Research on Technology of AUV Integrated Navigation System Based on Multivariate Information Fusion. Master’s Thesis.

[4] Wang L., Liu Y.-H., Wan J.-W., Shao J.-X. (2007). Multi-robot cooperative localization based on relative bearing. Chin. J. Sens. Actuators.

[5] Zhang F.-B., Zhang X.-L., Ma P. (2013). An algorithm of multi-AUVs cooperative location considering clock synchronization. Torpedo Technol..

[6] Li W.-B., Liu M.-Y., Lei X.-K., Pei X. (2011). Cooperative navigation for multiple autonomous underwater vehicles with single leader in unknown ocean current. Acta Armamentarii.

[7] Etter P.C. (2003). Underwater Acoustic Modeling and Simulation.

[8] Poursheikhali S., Zamiri-Jafarian H. TDOA based target localization in inhomogeneous underwater wireless sensor network. Proceedings of the 5th International Conference on Computer and Knowledge Engineering (ICCKE).

[9] Baccou P., Jouvencel B., Creuze V. Single beacon acoustic for AUV navigation. Proceedings of the International Conference on Advanced Robotics.

[10] Fang X.-P., Yan W.-S. (2012). Formation optimization for cooperative localization based on moving long baseline with two leader AUVs. Acta Armamentarii.

[11] Fang X.-P., Zhang F.-B., Li J.-B. (2014). Formation Geometry of Underwater Positioning Based on Multiple USV/AUV. Syst. Eng. Electron..

[12] Moreno-Salinas D., Pascoal A.M., Aranda J. (2011). Optimal Sensor Placement for Multiple Underwater Target Localization with Acoustic Range Measurements. IFAC Proc. Vol..

[13] Moreno-Salinas D., Pascoal A.M., Aranda J. Optimal Sensor Placement for Underwater Positioning with Uncertainty in the Target Location. Proceedings of the International Conference on Robotics and Automation.

[14] Moreno-Salinas D., Pascoal A.M., Almansa J.A. (2013). Optimal Sensor Placement for Multiple Target Positioning with Range-Only Measurements in Two-Dimensional Scenarios. Sensors.

[15] Moreno-Salinas D., Pascoal A.M., Almansa J.A. (2013). Sensor networks for optimal target localization with bearing-only measurements in constrained three dimensional scenarios. Sensors.

[16] Moreno-Salinas D., Pascoal A.M., Aranda J. (2013). Underwater Target Positioning with a Single Acoustic Sensor. IFAC Proc. Vol..

[17] Moreno-Salinas D., Pascoal A.M., Aranda J. (2016). Optimal Sensor Placement for Acoustic Underwater Target Positioning with Range-Only Measurements. IEEE J. Ocean. Eng..

[18] Doğançay K., Hmam H. On optimal sensor placement for time-difference-of-arrival localization utilizing uncertainty minimization. Proceedings of the 17th European Signal Processing Conference.

[19] Hamdollahzadeh M., Adelipour S., Behnia F. Recursive sensor placement in two dimensional TDOA based localization. Proceedings of the 24th Iranian Conference on Electrical Engineering (ICEE).

[20] Domingo-Pereza F., Lazaro-Galileaa J.L., Wieserb A., Martin-Gorostizaa E., Salido-Monzua D., de la Llanaa A. (2016). Sensor placement determination for range-difference positioning using evolutionary multi-objective optimization. Expert Syst. Appl..

[21] Hamdollahzadeh M., Adelipour S., Behnia F. Optimal sensor configuration for two dimensional source localization based on TDOA/FDOA measurements. Proceedings of the 17th International Radar Symposium (IRS).

[22] Meng W., Xie L., Xiao W. (2016). Optimal TDOA Sensor-Pair Placement with Uncertainty in Source Location. IEEE Trans. Veh. Technol..

[23] Ramezani H., Jamali-Rad H., Leus G. (2013). Target Localization and Tracking for an Isogradient Sound Speed Profile. IEEE Trans. Signal Process..

[24] Berger C.R., Zhou S., Willett P., Liu L. (2008). Stratification Effect Compensation for Improved Underwater Acoustic Ranging. IEEE Trans. Signal Process..

[25] Hovem J.M. (2013). Modeling and Measurement Methods for Acoustic Waves and for Acoustic Microdevices.

[26] Chan Y.T., Ho K.C. (1994). A simple and efficient estimator for hyperbolic location. IEEE Trans. Signal Process..

[27] Van Trees H.L. (2001). Detection, Estimation and Modulation Theory.

